# Detection of Vascular Mild Cognitive Impairment in Southeast Asia Using the Visual Cognitive Assessment Test: Machine Learning Analysis From the BIOCIS (Biomarkers and Cognition Study, Singapore)

**DOI:** 10.2196/76847

**Published:** 2025-10-08

**Authors:** Pricilia Tanoto, Hannah En Ye, Seyed Ehsan Saffari, Yi Jin Leow, Ashwati Vipin, Faith Phemie Hui En Lee, Smriti Ghildiyal, Shan Yao Liew, Adnan Azam Mohammed, Gurveen Kaur Sandhu, Kiirtaara Aravindhan, Gursimar Bhalla, Rasyiqah Binte Shaik Mohamed Salim, Nagaendran Kandiah

**Affiliations:** 1 Dementia Research Centre Singapore Lee Kong Chian School of Medicine Singapore Singapore; 2 School of Psychology University of Wollongong Wollongong Australia; 3 AI+ Neurology Division Duke-NUS Medical School Singapore Singapore; 4 Neuroscience and Mental Health Programme Lee Kong Chian School of Medicine Singapore Singapore; 5 National Healthcare Group Singapore Singapore

**Keywords:** vascular mild cognitive impairment, cognition, neuropsychological test, Southeast Asia, machine learning

## Abstract

**Background:**

Vascular mild cognitive impairment (VMCI) is a significant global health concern, particularly in Asia. The visual cognitive assessment test (VCAT) has shown promise as a language-neutral screening tool for cognitive impairment.

**Objective:**

This study aims to assess the effectiveness of the VCAT in detecting VMCI and compare its diagnostic performance with the widely used and validated Montreal Cognitive Assessment (MoCA).

**Methods:**

Cross-sectional data from 524 community-dwelling participants were analyzed from the BIOCIS (Biomarkers and Cognition Study, Singapore) and classified into cognitively unimpaired, non-VMCI, and VMCI groups. The participants underwent neuropsychological assessments and 3-T magnetic resonance imaging. The random forest technique and multivariable logistic regression were applied to assess the discriminative properties of the tests.

**Results:**

Participants with VMCI exhibited significantly lower performance across various neuropsychological tests (*P*<.001) and higher rates of vascular risk factors (*P*<.001). At a cutoff of 27, the VCAT achieved near-perfect accuracy in discriminating the VMCI group from the cognitively unimpaired group (area under the receiver operating characteristic curve=1; sensitivity=1; specificity=0.991). For differentiating the VMCI group from the non-VMCI group, both the VCAT and the MoCA showed optimal performance at a cutoff of 25 (area under the receiver operating characteristic curve=1.00; sensitivity=1.00; specificity=1.00).

**Conclusions:**

The VCAT could be a valuable tool for detecting VMCI, particularly in diverse, multilingual populations. Its comparable or even superior performance to the MoCA, combined with its language-neutral design, positions the VCAT as a strong addition to cognitive assessment toolkits for VMCI. However, the complex nature of cognitive processing in VMCI suggests that a multifaceted approach that integrates both visual and verbal assessments may ultimately offer the most comprehensive evaluation.

**International Registered Report Identifier (IRRID):**

RR2-10.14283/jpad.2024.89

## Introduction

### Background

Vascular dementia is the second most prevalent form of dementia globally, including in Singapore and across Asia [1-3]. However, pure vascular dementia is relatively rare and often presents alongside other comorbidities that may worsen cognitive symptoms and accelerate decline [4]. To address this complexity, the term *vascular cognitive impairment* has emerged as an umbrella concept. This term encompasses a wide range of cognitive deficits, attributed partially or wholly to vascular causes, including vascular risk factors and cerebrovascular disease [5]. Within this spectrum, vascular mild cognitive impairment (VMCI) specifically denotes the early, mild stage of cognitive decline due to vascular causes [6]. Early recognition of VMCI is crucial for timely intervention and management.

A key contributor to VMCI is cerebral small vessel disease (CSVD), which frequently coexists with neurodegenerative disorders, such as Alzheimer disease and Parkinson disease. This overlap intensifies cognitive deficits, physical disabilities, and other symptoms of neurodegeneration [7]. Notably, individuals with moderate to severe CSVD tend to experience faster cognitive decline compared to those without such burdens [8]. Furthermore, patients who have experienced a stroke are at heightened risk of developing dementia within months to a year, primarily due to cumulative cerebral vascular damage from recurrent strokes [9].

To date, the gold standard for diagnosing VMCI involves combining neuroimaging techniques to detect CSVD [10,11]. However, the clinical utility of neuroimaging is often constrained by higher costs and limited accessibility, particularly in resource-constrained settings [10,11]. Existing cognitive screening tools, such as the Mini-Mental State Examination and the Montreal Cognitive Assessment (MoCA), are widely validated but were only designed to detect general cognitive changes rather than the specific symptoms of VMCI [12]. In addition, these tests pose challenges in multilingual settings, such as the Asia-Pacific region. Moreover, their language-specific designs usually require multiple translations and validations, potentially introducing cultural and linguistic biases [13,14].

Therefore, the visual cognitive assessment test (VCAT) is emerging as a promising, language-neutral alternative for global cognitive screening. As a visually based global tool, the VCAT eliminates the need for translations, thereby minimizing linguistic biases [15,16]. Although it is designed to be a global cognition test, it potentially offers a comprehensive method for detecting VMCI by assessing key cognitive domains, such as processing speed and executive function. These domains are closely linked to visual skills, as visual information is processed in the brain to guide accurate actions and judgments [17]. In addition, the VCAT also covers a broad spectrum of cognitive abilities, including attention, memory, language, and visuospatial abilities, and social cognition [15]. Previous research indicates that patients with VMCI, compared to healthy controls, show pronounced impairment in processing speed and executive functioning, further highlighting the potential utility of VCAT as a screening tool for VMCI [18]. While previous studies have assessed the validity of VCAT in detecting early dementia [19,20], its specific application and validation in detecting mild cognitive impairment (MCI) related to VMCI have not been extensively explored. This gap in knowledge presents an opportunity to evaluate the effectiveness of the VCAT in a critical area of cognitive health.

### Objectives

The objectives of this study are twofold: first, to evaluate the utility of the VCAT in detecting VMCI and second, to determine the optimal cutoff points compared to other global cognitive screening tools for the identification of VMCI.

We hypothesize that the VCAT would be sensitive in detecting cognitive impairment in the setting of VMCI and that it is a reliable screening tool for VMCI. If validated, the VCAT could potentially provide clinicians with a more accessible and culturally flexible tool for early VMCI detection, facilitating timely interventions and improved patient outcomes across diverse populations.

## Methods

### Study Design and Participants

Participants were recruited from the local community as part of the BIOCIS (Biomarkers and Cognition Study, Singapore) conducted from February 2022 to February 2024. All participants were literate in English or Mandarin and had not experienced any major neurological, systemic, or psychiatric disorders within the past 2 years. Demographic data were collected through self-report questionnaires. Further details on the protocol can be found in the study conducted by Leow et al [21].

### Ethical Considerations

This study was approved by the Nanyang Technological University Institutional Review Board (2021-1036). Informed consent was obtained from all participants according to the Declaration of Helsinki and local clinical research regulations, and the procedures used in this study were in accordance with ethical guidelines.

### Neuropsychological Tests

Participants underwent a comprehensive set of neuropsychological tests, assessing global cognition (the MoCA and the VCAT), episodic memory (the Wechsler Memory Scale, fourth edition logical story delayed), executive function (the Trail Making Test Part B [TMT-B] and the Color Trails Test 2 [CT2]), visuospatial ability (the Wechsler Adult Intelligence Scale, fourth edition block design and the Rey-Osterrieth complex figure test copy), processing speed (the symbol digit modalities test), and language ability (semantic fluency [animal] from the MoCA test).

### Neuroimaging

Magnetic resonance imaging (MRI) scans were performed using a 3-T Prisma fit scanner (Siemens). MRI visual ratings were conducted on T1-weighted magnetization-prepared rapid gradient echo and T2-weighted fluid-attenuated inversion recovery sequences to quantify the Staals score (0-4). White matter hyperintensities (WMHs) volume was calculated using the *Computational Anatomy Toolbox 12*, an extension of Statistical Parametric Mapping within the MATLAB (MathWorks) environment.

### Classification of Participants

Cognitive classification was based on the criteria described by Petersen [22] and the National Institute on Aging–Alzheimer’s Association criteria [23]. Participants without subjective cognitive concerns and without impairment in cognitive tests (defined as scores of 1.5 SDs below the mean) were classified as cognitively unimpaired. Participants with subjective cognitive concerns and impaired cognitive test scores but without functional impairment were classified as having MCI. CSVD was determined using the Staals total small vessel disease score of 1 or more [24]. Consequently, participants were grouped into the cognitively unimpaired (CU), non-VMCI (NVMCI), and VMCI groups.

### Statistical Analysis

Continuous variables were reported as means and SDs, while categorical variables were presented as frequencies and percentages. Group comparisons of demographic and cognitive scores were conducted using the chi-square test (or the Fisher exact test, where appropriate) and ANOVA (or the Kruskal-Wallis test, depending on the normality assumption) for categorical and continuous variables, respectively. The discriminative properties of the VCAT and the MoCA were assessed using the random forest (RF) technique as part of the machine learning methodology. A total of 100 trees were run to train the RF model, where at most 4 variables were randomly selected as candidates at each split. A 10-fold cross validation (CV) was performed to avoid overfitting and increase the generalization accuracy of the model. The area under the receiver operating characteristic (ROC) curve (AUC) was calculated, along with 95% CI computed using 2000 stratified bootstrap replicates. Sensitivity and specificity metrics were calculated for various threshold points, and the exact binomial distribution approach was used to calculate the corresponding 95% CI. Prespecified pairwise group comparisons were performed and adjusted for clinical confounders, including age, sex, and years of education. *P* values were corrected using the Tukey honestly significant difference post hoc test. Vascular risk factors (eg, hypertension and hyperlipidemia) and their corresponding medications were not adjusted for, as group classification was based on MRI evidence of cerebrovascular disease rather than the presence or absence of such clinical risk factors. This rationale aligns with our imaging-based definition of the VMCI group. All statistical analyses were performed using R (R Foundation for Statistical Computing) [25].

## Results

### Participant and Cognitive Test Characteristics

A total of 524 participants were enrolled in this study. The cohort had a mean age of 59.05 (SD 11.33) years, with 199 (37.9%) being male, and a mean education duration of 14.45 (SD 3.61) years. Among the participants, 211 (40.3%) were classified as the CU group, 92 (17.6%) as the NVMCI group, and 221 (42.2%) as the VMCI group as shown in Table 1. Table 1 illustrates the demographics and vascular risks of the participants. The VMCI group exhibited significantly higher rates of hypertension (106/221, 47.9%) and diabetes mellitus (39/221, 17.6%) diagnoses compared to the CU and NVMCI groups. Furthermore, the VCMI group demonstrated a larger volume of WMHs. However, there were no discernible differences in BMI status or the incidence of hyperlipidemia across the groups.

**Table 1:** Demographic and vascular risk characteristics (N=524). Italics indicate significant values.

Characteristic	Overall	CU^a^ (n=211)	NVMCI^b^ (n=92)	VMCI^c^ (n=221)	*P* value^d^
Age (y), mean (SD)	59.05 (11.33)	52.18 (10.33)	58.12 (9.83)	65.91 (8.43)	*<.001*
Education (y), mean (SD)	14.45 (3.61)	15.41 (3.03)	14.60 (3.79)	13.48 (3.81)	*<.001*
Gender: male, n (%)	199 (38)	72 (34.1)	34 (37)	93 (42.1)	.30
BMI, mean (SD)	23.50 (3.34)	23.30 (3.32)	23.57 (3.43)	23.67 (3.33)	.30
Hypertension, n (%)	159 (30.3)	33 (15.6)	20 (21.7)	106 (48)	*<.001*
Diabetes mellitus, n (%)	53 (10.1)	8 (3.8)	6 (6.5)	39 (17.6)	*<.001*
Hyperlipidemia, n (%)	331 (63.2)	125 (59.2)	58 (63)	148 (67)	.20
White matter hyperintensities volume, mean (SD)	2.14 (3.13)	1.04 (0.87)	1.14 (0.66)	3.69 (4.36)	*<.001*

^a^CU: cognitively unimpaired.

^b^NVMCI: nonvascular mild cognitive impairment.

^c^VMCI: vascular mild cognitive impairment.

^d^*P* values were derived from ANOVA or the Kruskal-Wallis test for continuous variables and from the chi-square test or Fisher exact test for categorical variables.

### ROC Analysis of VCAT in Detecting VMCI

To evaluate the performance of the VCAT and the MoCA in detecting VMCI, we conducted ROC analyses using RF classifiers with 10-fold CV. As summarized in Table 2 both tools showed comparable diagnostic accuracy at the same cutoff score of 27 or less: MoCA (AUC=0.98; sensitivity=0.97; specificity=0.92) and VCAT (AUC=0.98; sensitivity=0.93; specificity=0.93). While this analysis was not the main focus of this study, it provided supportive evidence for the discriminative utility of both instruments.

ROC analysis using RF and 10-fold CV showed that the VCAT outperformed the MoCA in distinguishing VMCI from CU, as presented in Multimedia Appendix 1. At cutoff points of 25 and 26, the sensitivity of VCAT (0.94, 95% CI 0.90-0.96 and 0.92, 95% CI 0.88-0.95, respectively) was comparable to that of MoCA (0.95, 95% CI 0.91-0.97 for both), with overlapping CIs. Notably, at a cutoff of 27, the VCAT achieved perfect accuracy in identifying VMCI (AUC=1; sensitivity=1; specificity=0.99), surpassing the performance of the MoCA (AUC=0.99; sensitivity=0.96; and specificity=0.95) as shown in Table 2. The multivariable logistic regression yielded a comparable trend, further validating our findings.

We performed an ROC analysis to distinguish between NVMCI and VMCI (Table 3). The RF results showed that the VCAT outperformed the MoCA at all 3 cutoff points in detecting VMCI from the NVMCI. This was further supported by a 10-fold CV, where the VCAT (AUC=0.99) slightly exceeded the performance of the MoCA (AUC=0.99) at cutoff points of 26 and 27, respectively. At a cutoff point of 25, both the VCAT and the MoCA achieved perfect accuracy (AUC=1.00; sensitivity=1.00; specificity=1.00) in distinguishing NVMCI from VMCI, indicating that 25 was the optimal cutoff point for differentiating between the 2 groups as shown in Table 3.

**Table 2:** Discriminative properties of Montreal Cognitive Assessment (MoCA) and visual cognitive assessment test (VCAT) in detecting vascular mild cognitive impairment (VMCI) from cognitively unimpaired (CU).

Test and cutoff point		Analysis	Training				10-fold CV^a^		
			AUC^b^ (95% CI)	Sensitivity (95% CI)	Specificity (95% CI)	AUC (95% CI)		Sensitivity (95% CI)	Specificity (95% CI)
**MoCA**									
	**<25**								
		RF^c^	0.90 (0.87-0.93)	0.86 (0.81-0.91)	0.77 (0.71-0.83)	0.99 (0.99-0.99)		0.95 (0.91-0.97)	0.97 (0.93-0.98)
		Multivariable logistic regression	0.87 (0.83-0.90)	0.84 (0.78-0.88)	0.75 (0.69-0.81)	0.87 (0.83-0.90)		0.84 (0.78-0.88)	0.75 (0.69-0.81)
	**<26**								
		RF	0.90 (0.88-0.93)	0.83 (0.77-0.87)	0.80 (0.74-0.85)	0.99 (0.98-0.99)		0.95 (0.91-0.97)	0.95 (0.91-0.97)
		Multivariable logistic regression	0.86 (0.83-0.89)	0.84 (0.792-0.89)	0.74 (0.68-0.80)	0.86 (0.83-0.89)		0.84 (0.79-0.89)	0.74 (0.68-0.80)
	**<27**								
		RF	0.90 (0.88-0.93)	0.85 (0.79-0.89)	0.79 (0.73-0.84)	0.99 (0.99-0.99)		0.96 (0.93-0.98)	0.95 (0.92-0.98)
		Multivariable logistic regression	0.86 (0.83-0.90)	0.76 (0.70-0.81)	0.84 (0.78-0.89)	0.86 (0.83-0.90)		0.76 (0.70-0.81)	0.84 (0.78-0.89)
**VCAT**									
	**<25**								
		RF	0.91 (0.88-0.93)	0.86 (0.81-0.90)	0.79 (0.73-0.84)	0.99 (0.98-0.99)		0.94 (0.90-0.96)	0.95 (0.92-0.98)
		Multivariable logistic regression	0.86 (0.83-0.89)	0.82 (0.76-0.87)	0.80 (0.74-0.85)	0.86 (0.83-0.89)		0.82 (0.76-0.87)	0.80 (0.74-0.85)
	**<26**								
		RF	0.90 (0.88-0.93)	0.87 (0.82-0.91)	0.78 (0.72-0.84)	0.99 (0.98-0.99)		0.92 (0.88-0.95)	0.98 (0.95-0.99)
		Multivariable logistic regression	0.86 (0.83-0.89)	0.84 (0.79-0.89)	0.77 (0.71-0.83)	0.86 (0.83-0.89)		0.84 (0.79-0.89)	0.84 (0.79-0.89)
	**<27**								
		RF	0.90 (0.87-0.93)	0.83 (0.78-0.88)	0.80 (0.74-0.85)	1.00 (1.00-1)		1.00 (0.98-1.00)	0.99 (0.96-0.99)
		Multivariable logistic regression	0.86 (0.83-0.89)	0.86 (0.80-0.90)	0.75 (0.69-0.81)	0.86 (0.83-0.89)		0.86 (0.80-0.90)	0.75 (0.69-0.81)

^a^CV: cross validation.

^b^AUC: area under the curve.

^c^RF: random forest.

**Table 3:** Discriminative properties of Montreal Cognitive Assessment (MoCA) and visual cognitive assessment test (VCAT) in detecting vascular mild cognitive impairment (VMCI) from nonvascular mild cognitive impairment (NVMCI).

Test and cutoff point		Analysis	Training				10-fold CV^a^		
			AUC^b^ (95% CI)	Sensitivity (95% CI)	Specificity (95% CI)	AUC (95% CI)		Sensitivity (95% CI)	Specificity (95% CI)
**MoCA**									
	**>25**								
		RF^c^	0.85 (0.80-0.89)	0.85 (0.79-0.89)	0.69 (0.58-0.78)	1.00 (1.00-1.00)		1.00 (0.98-1.00)	1.00 (0.95-1.00)
		Multivariable logistic regression	0.74 (0.68-0.80)	0.79 (0.72-0.84)	0.60 (0.42-0.70)	0.74 (0.68-0.80)		0.70 (0.72-0.84)	0.60 (0.49-0.70)
	**>26**								
		RF	0.84 (0.79-0.88)	0.75 (0.68-0.80)	0.76 (0.65-0.84)	0.99 (0.99-0.99)		0.94 (0.90-0.94)	0.97 (0.92-0.99)
		Multivariable logistic regression	0.73 (0.67-0.80)	0.82 (0.76-0.87)	0.56 (0.45-0.67)	0.73 (0.67-0.80)		0.82 (0.76-0.87)	0.56 (0.48-0.67)
	**>27**								
		RF	0.85 (0.81-0.90)	0.72 (0.6-0.78)	0.81 (0.72-0.89)	0.99 (0.98-0.99)		0.96 (0.92-0.98)	0.96 (0.90-0.93)
		Multivariable logistic regression	0.74 (0.68-0.80)	0.57 (0.51-0.64)	0.79 (0.69-0.87)	0.74 (0.68-0.80)		0.57 (0.51-0.64)	0.79 (0.69-0.87)
**VCAT**									
	**>25**								
		RF	0.85 (0.80-0.89)	0.85 (0.80-0.89)	0.70 (0.59-0.79)	1.00 (1.00-1.00)		1.00 (0.98-1.00)	1.00 (0.95-1.00)
		Multivariable logistic regression	0.73 (0.67-0.80)	0.82 (0.77-0.87)	0.56 (0.45-0.67)	0.73 (0.67-0.80)		0.82 (0.77-0.87)	0.56 (0.45-0.67)
	**>26**								
		RF	0.85 (0.80-0.90)	0.72 (0.65-0.78)	0.79 (0.69-0.87	0.99 (0.99-1.00)		0.96 (0.93-0.98)	0.98 (0.93-1.00)
		Multivariable logistic regression	0.73 (0.67-0.79)	0.83 (0.77-0.87)	0.54 (0.46-0.65)	0.73 (0.67-0.79)		0.83 (0.77-0.87)	0.54 (0.43-0.65)
	**>27**								
		RF	0.82 (0.80-0.89)	0.78 (0.72-0.88)	0.72 (0.62-0.81)	0.99 (0.99-1.00)		0.97 (0.94-0.92)	0.98 (0.93-1.00)
		Multivariable logistic regression	0.73 (0.66-0.79)	0.79 (0.72-0.84)	0.60 (0.49-0.70)	0.73 (0.66-0.79)		0.70 (0.72-0.82)	0.60 (0.42-0.70)

^a^CV: cross validation.

^b^AUC: area under the curve.

^c^RF: random forest.

### Cognitive Performance

The analysis of covariance (ANCOVA revealed significant mean differences among the 3 groups based on neuropsychological tests, with the VMCI group performing lower than the CU and NVMCI groups after controlling for age, years of education, and sex (Table 4). However, this pattern was not consistent across all tests, particularly for the Wechsler Adult Intelligence Scale, fourth edition block design and the symbol digit modalities test assessments as in Table 4.

Further examination through pairwise comparisons using the Tukey honestly significant difference post hoc test indicated significant differences between NVMCI and VMCI groups in the VCAT, the MoCA, the CT2, the TMT-B, and semantic fluency (animals; *P*<.05). This suggested that the VCAT may be particularly effective in distinguishing between NVMCI and VMCI when compared to other global cognition tests, such as the MoCA, and domain-specific assessments, such as the CT2, the TMT-B, and semantic fluency.

**Table 4:** Mean differences of neuropsychological tests across 3 groups (N=524). Italics indicate significant values (*P*<.05).

Characteristic	Overall, mean (SD)	CU^a^ (n=211), mean (SD)	NVMCI^b^ (n=92), mean (SD)	VMCI^c^ (n=221), mean (SD)	*P* value^d^	NVMCI vs VMCI^d^	CU vs VMCI^d^
VCAT^e^	26.79 (2.67)	27.83 (2.05)	26.98 (2.33)	25.71 (2.91)	*<.001*	*<.001*	*<.001*
MoCA^g^	25.73 (2.72)	26.74 (2.46)	25.59 (2.51)	24.82 (2.72)	*<.001*	*.04*	*<.001*
Logical story delayed	18.70 (3.97)	20.59 (2.66)	17.65 (4.66)	17.31 (3.99)	*<.001*	.74	*<.001*
Color Trails Test 2	94.31 (38.40)	78.86 (28.08)	93.59 (30.14)	109.41 (43.83)	*<.001*	*.001*	*<.001*
Trail Making Test Part B	75.76 (46.69)	54.31 (16.53)	77.57 (34.29)	96.26 (60.24)	*<.001*	*.001*	*<.001*
Block design	40.93 (11.18)	44.73 (11.39)	40.39 (10.75)	37.33 (9.89)	.06	.06	*<.001*
Rey-Osterrieth complex figure test	32.21 (3.64)	33.84 (1.79)	30.72 (4.12)	31.22 (4.15)	*<.001*	.46	*<.001*
Semantic fluency	18.53 (4.94)	20.52 (4.69)	18.32 (5.11)	16.72 (4.37)	*<.001*	*.02*	*<.001*
Symbol digit modalities test	70.97 (31.80)	77.52 (19.24)	72.33 (14.97)	64.13 (43.24)	.14	.08	*<.001*

^a^CU: cognitively unimpaired.

^b^NVMCI: nonvascular mild cognitive impairment.

^c^VMCI: vascular mild cognitive impairment.

^d^Adjusted for age, sex, and years of education.

^e^VCAT: visual cognitive assessment test.

^g^MoCA: Montreal Cognitive Assessment.

## Discussion

### Principal Findings

This study aimed to evaluate the utility of the VCAT in detecting VMCI, benchmarking its performance against the widely used MoCA. By comparing VMCI not only with healthy controls (CU group) but also with another form of MCI (ie, NVMCI), this study provides robust evidence of the diagnostic utility of the VCAT. The results demonstrate the capability of the VCAT to accurately distinguish VMCI from both healthy individuals and other MCI subtypes, reinforcing its specificity and clinical relevance. These findings support our hypothesis and underscore the potential of the VCAT as a targeted diagnostic tool for VMCI, offering significant implications for clinical practice, particularly in guiding early interventions and paving the way for future research in cognitive impairment diagnostics.

### VCAT Performance and Clinical Implications

Our results demonstrate that VCAT performed comparatively better than the MoCA in detecting VMCI, particularly at higher cutoff points.

Although not the primary focus of this study, our supplementary analysis provides additional insight into the performance of the VCAT and the MoCA in detecting NVMCI. Both tools demonstrated high diagnostic accuracy at a cutoff score of 27 or less, with identical AUC values and comparable sensitivity and specificity. These findings suggest that the VCAT performs on par with the well-established MoCA, consistent with previous validation studies [26,27]. This further supports the diagnostic utility of the VCAT as a viable alternative cognitive screening tool, particularly in contexts where language or cultural factors may limit the effectiveness of the MoCA.

With an optimal cutoff of 27, the VCAT showed excellent discriminative properties in distinguishing individuals with VMCI from individuals without vascular cognitive impairment (ie, the CU group). The high sensitivity and specificity found in our study suggest that the VCAT could be a valuable tool for the early detection of VMCI, potentially allowing for earlier intervention and management strategies. Notably, previous studies have identified similar cutoff scores (approximately 27) for detecting MCI in general older Southeast Asian populations [19]. Our findings extend this evidence by demonstrating that the same cutoff may also be effective in detecting vascular cognitive impairment within a comparable cohort. This supports the utility of the VCAT in vascular-specific contexts and underscores the need for continued refinement of its thresholds across different MCI subtypes.

Furthermore, the VCAT demonstrated promise in differentiating between the vascular etiologies in populations with MCI groups (ie, NVMCI and VMCI). With a cutoff score of 25, the VCAT showed comparable performance to the MoCA in this differentiation. While neither the MoCA nor the VCAT was specifically designed to detect VMCI, these findings suggest that both, especially the VCAT, can capture broader cognitive impairments that may be present regardless of etiology. This potential to distinguish between vascular and nonvascular cognitive impairment in participants in our cohort may help guide clinicians in tailoring interventions and follow-up care more effectively.

In our study analysis, we used RF, as it offers several key benefits that enhance its effectiveness. This method is a useful tool in clinical research [28,29], as it aggregates the predictions of multiple decision trees and provides high accuracy and robustness, making it particularly valuable for distinguishing between different cognitive states, such as VMCI, CU, and NVMCI. In addition, RF is less prone to overfitting compared to single decision trees, and it does not require extensive feature scaling, simplifying the data preprocessing steps [30]. Its robustness to noise and ability to handle missing data further contribute to reliable and insightful analysis, making RF a powerful tool for assessing and validating the discriminative properties of the VCAT.

The visual nature of the VCAT offers both advantages and potential limitations. Its language-independent approach makes it particularly valuable in diverse, multilingual populations where verbal tests may introduce linguistic biases. However, visual and verbal testing may use distinct pathways for processing semantic memory and implicit learning, emphasizing the importance of assessing both visual and verbal memory in cognitive evaluations, as highlighted in previous research [31].

Despite these limitations, the VCAT remains a valuable screening tool for VMCI. Its language-independent, visual approach addresses a key limitation of many existing cognitive assessment tools, which often require translation and may lose validity across different linguistic and cultural contexts [32].

### Cognitive and Vascular Profile of VMCI

Our findings reinforce the distinct cognitive profile associated with VMCI. The VMCI group demonstrated significantly lower performance across various neuropsychological tests compared to the CU and NVMCI groups. This underscores the importance of comprehensive cognitive assessment in suspected VMCI cases, as global cognition tests alone may not fully capture the specific impairments due to different underlying pathophysiological mechanisms.

In our study, the VMCI group showed lower MoCA scores compared to both the CU and NVMCI groups. This may suggest that individuals with VMCI are at a more advanced stage of cognitive impairment. However, it is also possible that the lower scores reflect the specific effects of cerebrovascular disease, which can particularly impact attention and executive function [33]—domains heavily weighted in the MoCA. Therefore, the reduced performance in the VMCI group may be due to both greater overall cognitive decline and the influence of vascular pathology on specific cognitive areas.

Notably, our study revealed significant differences in processing speed and executive functioning between the VMCI and NVMCI groups, consistent with previous studies [34,35]. Furthermore, we observed significant differences between the NVMCI and VMCI groups across other cognitive domains, such as episodic memory, visuospatial ability, and language, as reflected in logical story delayed, block design, and fluency tests. This supports the growing body of evidence suggesting that VMCI is not necessarily confined to a single domain and may present with more subtle or multifaceted cognitive changes [36-38]. These findings highlight the complexity of cognitive deficits in VMCI and point to the potential influence of comorbid pathology.

The higher prevalence of hypertension and diabetes mellitus in the VMCI group, along with larger volumes of WMHs, highlights the strong association between vascular risk factors and WMHs with cognitive impairment. These findings support our hypothesis and align with previous research [39,40]. They also highlight the potential for preventive strategies targeting vascular health in mitigating cognitive decline. This underscores the importance of considering vascular risk factors in the assessment and management of cognitive impairment.

### Clinical Implications

This study presents several important clinical implications. First, the VCAT demonstrates strong utility as a screening tool for VMCI, likely capturing broader cognitive impairments—particularly in processing speed and executive function—that are commonly affected in this population. Second, we established specific VCAT cutoff scores: 27 to distinguish VMCI from CU and 25 to differentiate VMCI from NVMCI. These cutoff points offer practical guidance for clinicians, enhancing accuracy and consistency in interpreting results. Third, the distinct cognitive profile observed in patients with VMCI, marked by deficits in processing speed and executive function, highlights that these key domains could be prime targets for early intervention. Addressing these specific deficits may improve patient outcomes and delay disease progression. Finally, the strong association between vascular risk factors and VMCI underscores the importance of aggressive management of vascular risk factors, both in reducing the incidence of VMCI and mitigating further cognitive decline.

### Limitations and Future Directions

While our study provides valuable insights, several limitations should be noted. While the VCAT demonstrates comparable or slightly superior performance to the MoCA, particularly at higher cutoff points, its reliance on visual processing may not provide a complete picture of cognitive function.

First, although we applied a comprehensive set of exclusion criteria, we cannot entirely rule out the presence of undiagnosed neurodegenerative conditions, such as Parkinsonism or other cognitive disorders, especially given our community-based recruitment. Subclinical or prodromal cases may not be detectable through standard structural MRI sequences used in this study, which lack the sensitivity to identify early-stage Parkinsonian pathology. Second, the VCAT may have potential bias toward visual processing at the expense of verbal abilities. As highlighted earlier, while this characteristic makes the VCAT valuable in diverse, multilingual populations by minimizing linguistic biases, it may not capture the full spectrum of cognitive deficits in individuals with stronger verbal abilities.

Therefore, future studies should aim to delineate the specific pathways involved in visual and verbal cognitive processing and investigate their relative contributions to VMCI. In addition, while the VCAT has proven to be an effective screening tool, the complex nature of cognitive processing in VMCI suggests that a multifaceted approach, incorporating both visual and verbal assessments, may ultimately offer the most comprehensive evaluation. As our understanding of these cognitive pathways evolves, this may lead to refined screening protocols that leverage the strengths of both visual and verbal testing modalities. The cross-sectional design limits our understanding of the VCAT’s predictive value for future cognitive decline. The sample size, particularly in the NVMCI group, was relatively small, which may have affected the statistical power of some comparisons, particularly when using the RF technique. The imbalanced nature of the datasets could lead to biased predictions favoring the majority, potentially affecting the model’s ability to accurately reflect the true predictive power. While we controlled for age, education, and sex, other potential confounding factors such as socioeconomic status or lifestyle factors were not accounted for. Furthermore, this study was conducted in a single geographic location, which may limit the generalizability of our findings. Therefore, future research should be conducted with longitudinal, larger, and more balanced samples that could provide more robust results.

### Conclusions

In conclusion, our study demonstrates the potential of the VCAT as a valuable tool for detecting VMCI, particularly in diverse, multilingual populations. Its performance, which is comparable with or superior to established tools such as the MoCA, combined with its language-neutral design, positions the VCAT as a significant addition to the cognitive assessment toolkit. However, the complexity of cognitive processing in VMCI suggests that a multifaceted approach, incorporating both visual and verbal assessments, may offer the most comprehensive evaluation.

Given the growing global burden of vascular cognitive impairment, tools such as the VCAT could be pivotal in early detection and intervention, potentially improving outcomes for individuals affected by these conditions. Future research should prioritize longitudinal validation, cross-cultural applicability, and integration into clinical practice guidelines for cognitive impairment assessment.

## Appendix

Multimedia Appendix 1 Discriminative properties of Montreal Cognitive Assessment and visual cognitive assessment testing in differentiating vascular mild cognitive impairment from cognitive unimpairment.

## Abbreviations

AUC: area under the receiver operating characteristic curve
BIOCIS: Biomarkers and Cognition Study, Singapore
CSVD: cerebral small vessel disease
CT2: Color Trails Test 2
CV: cross validation
MCI: mild cognitive impairment
MoCA: Montreal Cognitive Assessment
MRI: magnetic resonance imaging
CU: cognitively unimpaired
NVMCI: nonvascular mild cognitive impairment
RF: random forest
ROC: receiver operating characteristic
TMT-B: Trail Making Test Part B
VCAT: visual cognitive assessment test
VMCI: vascular mild cognitive impairment
WMH: white matter hyperintensity
