# Supplementary material for: Detection of Vascular Mild Cognitive Impairment in Southeast Asia Using the Visual Cognitive Assessment Test: Machine Learning Analysis From the BIOCIS (Biomarkers and Cognition Study, Singapore)
Source: JMIR Aging. 2025 Oct 8;8:e76847. doi: 10.2196/76847 (PMC12547338; doi:10.2196/76847)
Supplement: Multimedia Appendix 1 [file aging_v8i1e76847_app1.docx]

Table S1. Discriminative Properties of MoCA and VCAT in Detecting NVMCI from NVCU

| Cut-off points |  |  | Training | | | 10-fold CV | | |
| --- | --- | --- | --- | --- | --- | --- | --- | --- |
|  |  |  | AUC (CI) | Sens (CI) | Spec (CI) | AUC (CI) | Sens (CI) | Spec (CI) |
|  | | **Analysis** | **MoCA** | | | | | |
| <25 |  | a  b | 0.89 (0.86, 0.93)  0.69 (0.63, 0.75) | 0.75(0.64, 0.83)  0.77 (0.67, 0.85) | 0.88 (0.83, 0.92)  0.521(0.45, 0.59) | 0.98 (0.93, 0.99)  0.69 (0.63, 0.75) | 0.92 (0.85, 0.97)  0.77 (0.67, 0.85) | 0.95 (0.91, 0.97)  0.52 (0.45, 0.59) |
| <26 |  | a  b | 0.90 (0.86, 0.93)  0.68 (0.62, 0.75) | 0.89 (0.81, 0.95)  0.63 (0.52, 0.73) | 0.79 (0.72, 0.84)  0.68 (0.61, 0.74) | 0.98 (0.97, 0.99)  0.68 (0.62, 0.75) | 0.93 (0.86, 0.97)  0.63 (0.52, 0.73) | 0.92 (0.87, 0.95)  0.68 (0.61, 0.75) |
| <27 |  | a  b | 0.89 (0.86, 0.93)  0.67 (0.60, 0.74) | 0.85 (0.75, 0.91)  0.56 (0.45, 0.66) | 0.78 (0.71, 0.83)  0.74 (0.68, 0.80) | 0.98 (0.97, 0.99)  0.67 (0.60, 0.74) | 0.97 (0.91, 0.99)  0.56 (0.45, 0.66) | 0.92 (0.87, 0.95)  0.74 (0.68, 0.80) |
|  | |  | **VCAT** | | | | | |
| <25 |  | a  b | 0.88 (0.85, 0.92)  0.67 (0.60, 0.73) | 0.82 (0.73, 0.90)  0.69 (0.59, 0.79) | 0.77 (0.71, 0.83)  0.56 (0.49, 0.63) | 0.98 (0.97, 0.99)  0.67 (0.60, 0.73) | 0.92 (0.85, 0.97)  0.69 (0.59, 0.79) | 0.94 (0.90, 0.97)  0.56 (0.49, 0.63) |
| <26 |  | a  b | 0.88 (0.85, 0.92)  0.66 (0.60, 0.73) | 0.88 (0.79, 0.94)  0.43 (0.32, 0.54) | 0.73 (0.67, 0.79)  0.83 (0.78, 0.88) | 0.98 (0.97, 0.99)  0.66 (0.60, 0.73) | 0.92 (0.85, 0.97)  0.43 (0.32, 0.54) | 0.92 (0.88, 0.96)  0.83 (0.78, 0.88) |
| <27 |  | a  b | 0.88 (0.84, 0.92)  0.67 (0.60, 0.73) | 0.85 (0.75, 0.91)  0.48 (0.38, 0.59) | 0.74 (0.68, 0.80)  0.80 (0.73, 0.85) | 0.98 (0.97, 0.99)  0.67 (0.60, 0.73) | 0.93 (0.86, 0.97)  0.48 (0.38, 0.59) | 0.93 (0.89, 0.96)  0.80 (0.73, 85) |

*Note.* a = Random forest, b = Multivariable Logistic Regression

*Abbreviations.* 10-fold CV = 10-fold Cross Validation, AUC = Area Under the Curve, CI = Confidence Interval, MoCA = Montreal Cognitive Assessment, Sens = Sensitivity, Spec = Specificity, VCAT = Visual Cognitive Assessment Test.
